# Reconstruction of a ruptured patellar tendon using ipsilateral semitendinosus and gracilis tendons with preserved distal insertions: two case reports

**DOI:** 10.1186/1756-0500-6-361

**Published:** 2013-09-08

**Authors:** Yuji Takazawa, Hiroshi Ikeda, Muneaki Ishijima, Mitsuaki Kubota, Yoshitomo Saita, Haruka Kaneko, Yohei Kobayashi, Ryo Sadatsuki, Shinnosuke Hada, Kazuo Kaneko

**Affiliations:** 1 Department of Orthopaedics, Juntendo University School of Medicine, 2-1-1 Hongo, Bunkyou-ku, Tokyo 113-8421, Japan

## Abstract

**Background:**

Acute patellar tendon ruptures with poor tissue quality. Ruptures that have been neglected are difficult to repair. Several surgical techniques for the repair of the patellar tendon have been reported, however, these techniques remain difficult because of contractures, adhesions, and atrophy of the quadriceps muscle after surgery.

**Case presentation:**

We report the cases of 2 Japanese patients (Case 1: a 16-year-old male and Case 2: a 43-year-old male) with patellar tendon ruptures who were treated by reconstruction using semitendinosus-gracilis (STG) tendons with preserved distal insertions. Retaining the original insertion of the STG appears to preserve its viability and provide the revascularization necessary to accelerate healing. Both tendons were placed in front of the patella, in a figure-of-eight fashion, providing stability to the patella.

**Conclusion:**

Both patients recovered near normal strength and stability of the patellar tendon as well as restoration of function after the operation.

## Background

Patellar tendon ruptures are uncommon injuries that may become chronic or neglected if not correctly diagnosed [1]. The management goal of this type of injury is the restoration of the extensor mechanism that facilitates active knee extension. This can be accomplished by end-to-end sutures or reinsertion, usually combined with an additional reconstruction to protect the injured tendon [2]. Several reconstruction techniques have been proposed, including the use of synthetic material; autografts using the semitendinosus alone, or together with the gracilis tendon; the contralateral patellar tendon; turndown of the quadriceps tendon; or an allograft using the Achilles tendon [3-6]. However, the technique remains difficult to perform because of contractures, adhesions, and atrophy of the quadriceps muscle after surgery [7].

Here, we describe the cases of 2 patients with patellar tendon ruptures who were treated by reconstruction and restoration using semitendinosus–gracilis (STG) tendons with preserved distal insertions.

Surgery was performed using an anterior midline approach from the proximal pole of the patella to a position approximately 8 cm distal to the tibial tuberosity. After raising the flaps, scar tissues were excised and distal mobilization of the patella was performed. The remnants were then repaired with primary sutures, as far as possible.

**Figure 1:**
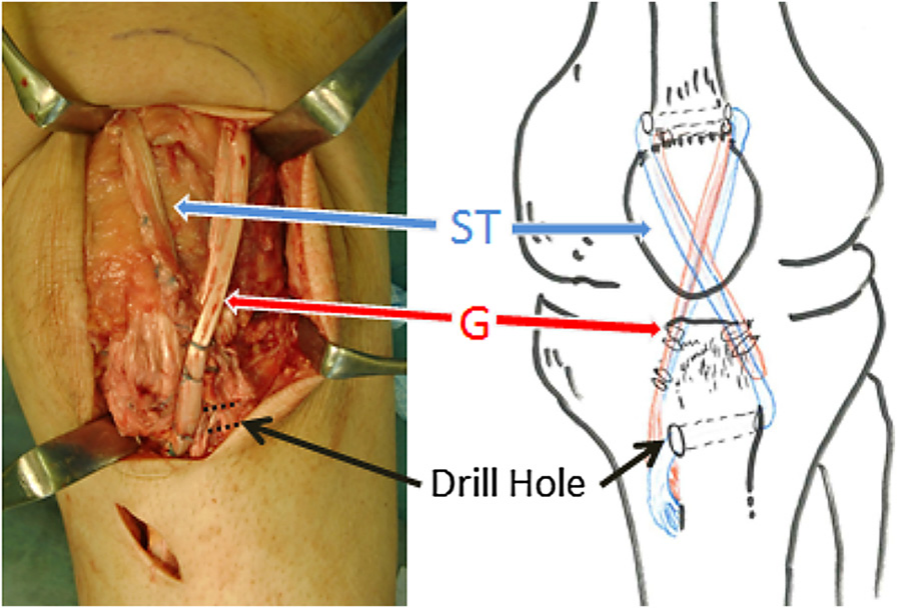
**Reconstruction of the patellar tendon using semitendinosus and gracilis tendons with preserved distal insertions.** The free end of the semitendinosus tendon was passed through a tunnel in the tibia tubercle in a medial to lateral direction. The free ends of both tendons were brought up proximally to cross in front of the patella as a figure-of-eight, towards the inferolateral side of the patella. Each tendon was passed transversely through the distal end of the quadriceps tendon and pulled distally. Both tendons were treated with interrupted sutures where they overlapped. ST: semitendinosus tendon, G: gracilis tendon.

The ends of the semitendinosus and gracilis tendons were exposed through another 4-cm incision over the surface of the pes anserinus. An open-ended tendon stripper was used to harvest the semitendinosus and gracilis tendons, with preservation of the tibial insertions. Both tendons were sutured along their longitudinal axes, after cleaning away any remaining muscle and fatty tissue. A horizontal tunnel, with a 4.5-mm diameter, was drilled in a medial to lateral direction at a level 1 cm posterior to the tibial tuberosity. The free end of the semitendinosus tendon was passed through this tunnel in a medial to lateral direction. Both tendons were then placed in front of the patella in a figure-of-eight fashion. The gracilis tendon was passed through the medial side of tibia, towards the inferolateral part of the patella, whereas the semitendinosus tendon was passed through the lateral side of tibia, towards the inferomedial part of the patella. Each tendon was then introduced, transversely, through the distal end of the quadriceps tendon along the proximal margin of the patella. The 2 tendons were pulled distally and placed in a figure-of-eight fashion, again. Finally, the tendons were sutured with interrupted sutures where they overlapped (Figure 1). The tension was set in such a manner that the lengths of the reconstructed tendons were approximately equal to the height of the patella. This distance conforms to the Insall–Salvati ratio [8] and standard postoperative radiographs were obtained to confirm the patellar height. The residual tissue between the patella and the tubercle tibia was sutured with overlapping sutures.

After the surgery, the knee was not splinted, but continuous passive motion training and isometric quadriceps exercise were gradually initiated on Day 1. Non-weight-bearing ambulation, using 2 crutches for balance, was initiated within 2 days after the operation. Partial weight-bearing walking was permitted, beginning 2 weeks after the surgery, with use of an extension brace. The brace was removed and closed kinetic chain exercises were performed, starting at 4 weeks after the operation. Jogging was encouraged after 3 months, and open kinetic chain exercises were allowed 4 months after the surgery. Sprinting and various competitive exercises were permitted 6 months after the operation and the patient was allowed to return to full sports activities, in a step-by-step manner, starting 8 months after the surgery.

## Case presentation

### Case 1

**Figure 2:**
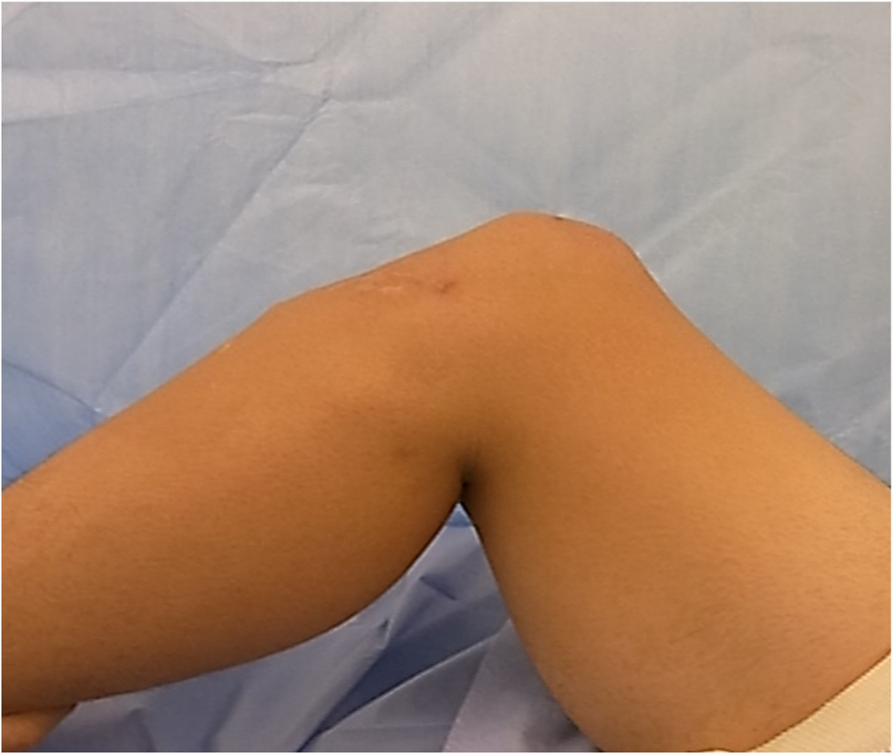
Lateral view of case 1 shows a defect located below the left patella and the proximal patellar position.

**Figure 3:**
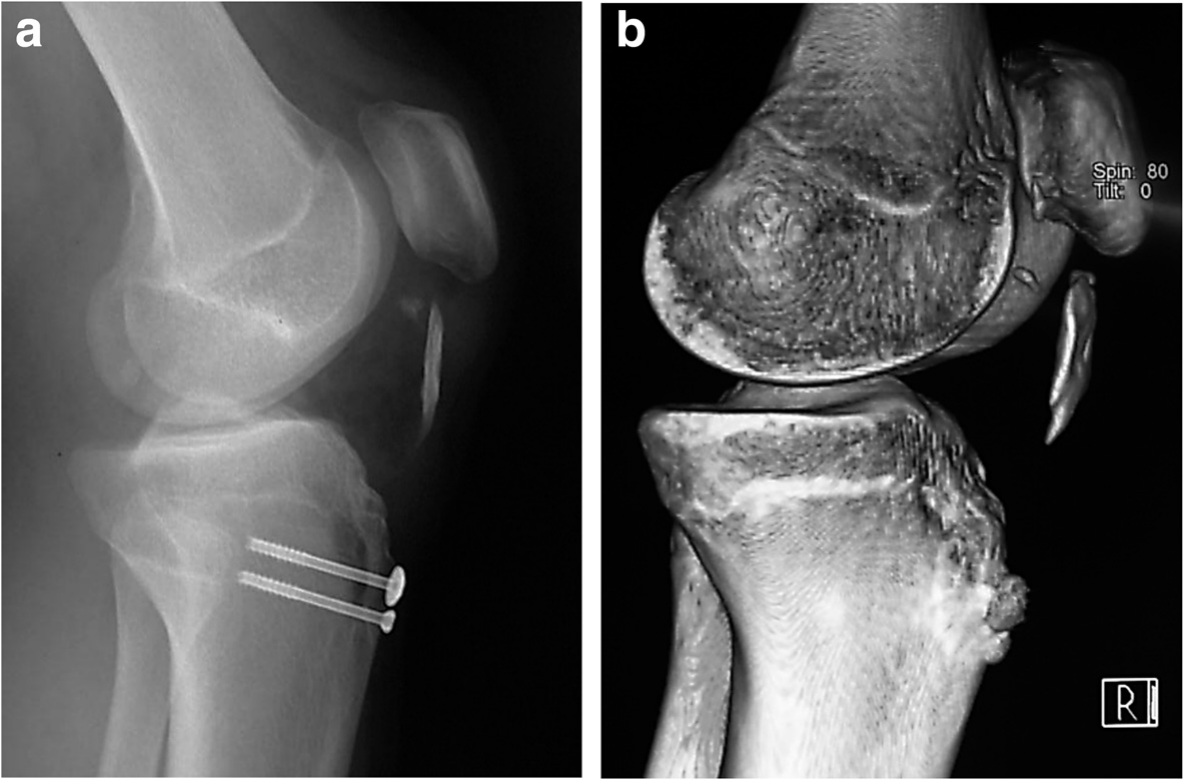
Lateral radiography (a) and computed tomography with three-dimensional reconstruction (b) of the right knee shows the presence of patella alta (Insall-Salvati index (Length of the patella/Length of the patella tendon) = 1.6) and ectopic calcification along the patellar tendon in case 1.

A 16-year-old male Japanese patient presented to our clinic with an inability to extend his left knee. He had injured his left knee during a jump while playing handball and sustained an avulsion fracture of the tibial tubercle Open reduction and cannulated screw fixation for the tibial plateau fracture had been performed at another institution 10 months before he visited our hospital. However, the rupture of the patellar tendon had been missed during the initial emergency treatment. He complained of patellar dislocation, an inability to run, and difficulty in walking up and down stairs. An examination indicated that his left patella had migrated proximally and was higher than the right (Figure 2). There was evidence of quadriceps wasting, and a palpable gap below the patella was noted. The range of movement was from 0 to 130°; however, there was no active knee extension. The preoperative Lysholm knee-score [9] was 65 points. Both plain radiography and computed tomography with three-dimensional reconstruction confirmed that there was massive ectopic calcification along the patellar tendon and severe patella alta (Insall-Salvati index, 1.6) (Figure 3). Magnetic resonance imaging indicated the absence of continuity in the patellar tendon and an irregularity in the tibial tuberosity.

During surgery, two-thirds of the patellar tendon mass was found to have been replaced by bone. The bony mass, which was 5.5 × 3.0 × 2.0 cm in size, was removed. Rupture of the patellar tendon from the inferior pole of the patella was identified during the operation. The semitendinosus and gracilis tendons were used to reconstruct the patellar tendon, as described. The ruptured patellar tendon remnant was sutured to strengthen the reconstruction, and the course of rehabilitation proceeded, as described. His knee functions gradually returned to normal, and no pain was reported. He was able to resume his sports activities 6 months after surgery and returned to full training 8 months after surgery. The patient was last examined 1 year after his operation. At that time, there was no extensor lag and full flexion could be achieved. Isokinetic peak torque measurements of quadriceps strength at 90°/s was only 5% less than that of the contralateral extremity, as confirmed by Biodex (Biodex Medical Systems Inc., New York, USA) testing. The Lysholm knee-score was 100 points.

### Case 2

**Figure 4:**
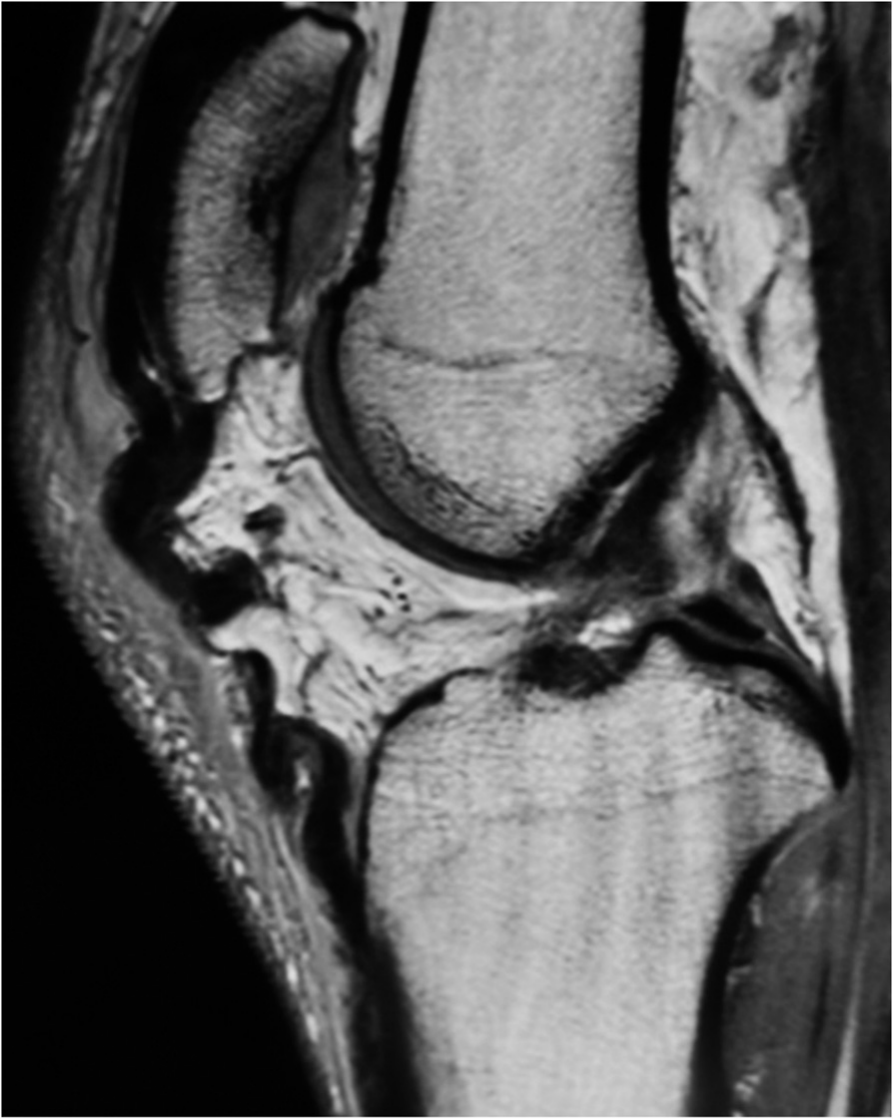
Proton magnetic resonance image (sagittal view) of case 2 demonstrating hemorrhage and focal discontinuity, consistent with a rupture of the left patellar tendon.

A 43-year-old male Japanese patient had injured his left knee during a jump while playing basketball. He had a history of rupturing both Achilles tendons and his body weight was 95 kg. A clinical examination revealed prominent pain in the patellar retinaculum, and lateral radiographs indicated the presence of patella alta (Insall index = 1.5). A diagnosis of patellar instability was made and surgical intervention was recommended. Proton magnetic resonance imaging revealed a rupture of the left patellar tendon (Figure 4).

**Figure 5:**
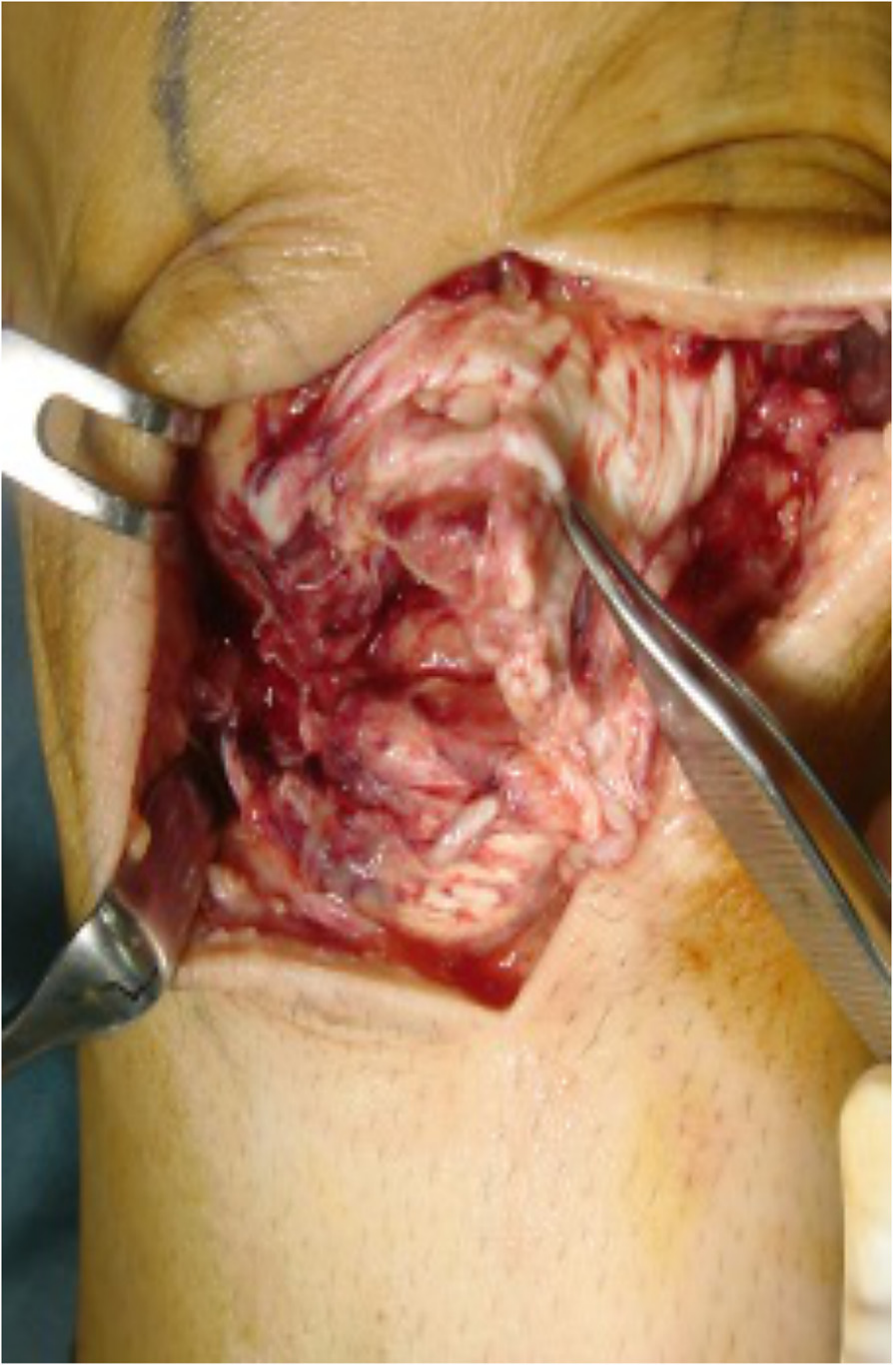
During surgery on case 2, the quality of ruptured patella tendon was poor and augmentation was needed.

During the surgery, which was performed 10 days after the injury, the remnants of the patellar tendons were observed to be thin and fragile. Those were repaired with primary sutures, as far as possible; however, the tissue quality was poor and augmentation was required (Figure 5). Reconstruction using semitendinosus and gracilis tendons with preserved distal insertion was performed and rehabilitation proceeded, as previously described. The patient returned to work as a technician 2 months after surgery. At the 1-year follow-up, he did not experience any pain or difficulty during his daily activities. Full flexion could be achieved and was comparable with the contralateral side; his quadriceps strength was marked as 5/5 and his thigh girth, 10 cm above the patella, was comparable to that of the right leg. The Lysholm knee-score was 95 points.

## Discussion

Fresh ruptures of the patellar tendon are relatively uncommon and require immediate surgical restoration of the extensor mechanism for optimal return to preinjury functional status. Ruptures are associated with tendinopathy caused by repetitive microtrauma, diabetes mellitus, rheumatoid arthritis, chronic renal failure, or steroid medication [10]. We reported 2 cases of patellar tendon disruption. One case involved a neglected disruption that had been missed during an initial emergency treatment. The second case involved an obese man who experienced a sports-related injury; poor quality of the ruptured tendon was suspected. Both cases required additional augmentation.

There are several surgical procedures for the repair of fresh and neglected ruptures of the patellar tendon; however, none of the methods are widely accepted. The treatment goals for ruptured patellar tendons include restoration of the quadriceps mechanism, restoration of the anatomic congruity of the patellofemoral joint to avoid chondral lesions, improved range of motion, and splinting of the patellar tendon to allow early mobilization. Early surgical treatment can yield optimal clinical results, and end-to-end repair or reinsertion is used, with or without cerclage reinforcement [2]. In cases involving poor tissue quality due to an underlying disease process or the severity of the injury, primary repair, combined with autogenous graft augmentation, has been considered.

Neglected rupture of the patellar tendon is a rare occurrence, the treatment of which is a difficult challenge for orthopedic surgeons. Use of a contralateral patellar tendon [5] may result in additional damage to the uninjured leg, whereas the use of synthetic materials [3] and allografts [6] increases the risk of bacterial or viral infection and neoplasia. We performed reconstruction and restoration of ruptured patellar tendons using STG tendons with preserved distal insertions, without the use of any synthetic materials. We suggest that this modified technique has several advantages. STG tendons are rich in tendon fibers and can be used to yield a strong graft; the ultimate tensile load of doubled semitendinosus tendon grafts have been reported to reach 2330 N [11] and they are often used in anterior cruciate ligament [ACL] reconstructions. The strength and stability of the extensor mechanism can also be restored with minimal donor site morbidity [4,12]. Preservation of the original distal insertion of the STG appears to preserve its viability and provide sufficient blood supply to accelerate healing. Chen et al. [13] reported the reconstruction of a ruptured patellar tendon using STG tendons with preserved distal insertions. However, in their technique, a combined fixation with wire was required. We did not use wire cerclage, and this may have reduced the risk of infection and obviated the need for a second intervention to remove the implanted material. Nyemb et al. [14] also described a reconstruction technique with a semitendinosus autograft that maintained its original insertion. However, their technique required the formation of a horizontal tunnel in the patella; such a hole in the small patella promotes a risk of fracture [15]. The presently described technique does not require the formation of a hole in the patella as both tendons were placed in front of the patella in a figure-of-eight fashion for additional stabilization. This technique is able to transmit the tension load from the patella directly to the tubercle tibia. When the knee bends, the patella is pulled downward and prevented from floating anteriorly by both tendons. In addition, this technique helps to stabilize the patella and maintain appropriate tracking. Most importantly, this technique avoids postoperative casting and facilitates immediate postoperative mobilization.

## Conclusion

In the two cases of patellar tendon ruptures that we describe here, we have tried to depict the usefulness of a technique that uses reconstruction using semitendinosus-gracilis (STG) tendons with preserved distal insertions. This procedure could be used for similar cases with acute patellar tendon ruptures involving poor tissue quality as well as for neglected ruptures because it provides good functional results, appropriate patellar alignment, and full active range of motion for the repaired knee. In addition, this technique may allow optimal healing of the repaired site and early knee mobilization.

## Consent

Written informed consent was obtained from the patient’s mother, in case of Case 1 and the patient himself for case 2 for publication of this case report and accompanying images. A copy of the written consent is available for review by the Editor-in-Chief of this journal.

## Competing interests

The authors declare that they have no competing interests.

## Authors’ contributions

YT (corresponding author) mainly performed medical examinations and surgeries for all patients. As assistant of operative procedure, YS and HK supported for case1 and HI and SH supported for case2. MI, MK, YK, RS and KK discussed and advised about the method of the operation. All authors read and approved the final manuscript.
